# The 372 T/C genetic polymorphism of TIMP-1 is associated with serum levels of TIMP-1 and survival in patients with severe sepsis

**DOI:** 10.1186/cc12739

**Published:** 2013-05-25

**Authors:** Leonardo Lorente, Mar Martín, Fátima Plasencia, Jordi Solé-Violán, José Blanquer, Lorenzo Labarta, César Díaz, Juan María Borreguero-León, Alejandro Jiménez, José Antonio Páramo, Josune Orbe, José A Rodríguez, Eduardo Salido

**Affiliations:** 1 Intensive Care Unit, Hospital Universitario de Canarias, Ofra s/n La Laguna 38320, Santa Cruz de Tenerife, Spain; 2 Intensive Care Unit, Hospital Universitario Nuestra Señora Candelaria, Carretera Rosario s/n, Santa Cruz Tenerife 38010, Spain; 3 Research Unit, Hospital Universitario de Canarias, La Laguna 38320, Santa Cruz de Tenerife, Spain; 4 Intensive Care Unit, Hospital Universitario Dr. Negrín, Barranco de la Ballena s/n, Las Palmas de Gran Canaria 35010, Spain; 5 Intensive Care Unit, Hospital Clínico Universitario de Valencia, Avenida Blasco Ibáñez nº17, Valencia 46004, Spain; 6 Intensive Care Unit, Hospital San Jorge de Huesca, Avenida Martínez de Velasco nº36, Huesca 22004, Spain; 7 Intensive Care Unit, Hospital Insular, Plaza Dr. Pasteur s/n, Las Palmas de Gran Canaria 35016, Spain; 8 Laboratory Deparment, Hospital Universitario de Canarias, Ofra s/n, La Laguna 38320, Santa Cruz de Tenerife, Spain; 9 Atherosclerosis Research Laboratory, CIMA-University of Navarra, Avenida Pío XII nº55, Pamplona 31008, Spain

**Keywords:** tissue inhibitor of matrix metalloproteinase-1, genetic, polymorphism, sepsis, mortality

## Abstract

**Introduction:**

Previous studies have found higher circulating levels of tissue inhibitor of matrix metalloproteinase (TIMP)-1 in nonsurviving septic patients than in surviving septic patients, and an association between the 372 T/C genetic polymorphism of TIMP-1 and the risk of developing certain diseases. However, the relationship between genetic polymorphisms of TIMP-1, circulating TIMP-1 levels and survival in patients with severe sepsis has not been examined, and this was the objective of the study.

**Methods:**

This multicentre, prospective, observational study was carried out in six Spanish ICUs. We determined the 372 T/C genetic polymorphism of TIMP-1 (rs4898), serum levels of TIMP-1, matrix metalloproteinase (MMP)-9, MMP-10, TNFα, IL-10 and plasma plasminogen activator inhibitor-1 (PAI-1). Survival at 30 days from ICU admission was the endpoint assessed. The association between continuous variables was carried out using Spearman's rank correlation coefficient or Spearman's rho coefficient. Multivariate logistic regression analysis was applied to determine the association between the 372 T/C genetic polymorphism and survival 30 days from ICU admission.

**Results:**

Of 275 patients with severe sepsis, 80 had genotype CC, 55 had genotype CT and 140 had genotype TT of the 372 T/C genetic polymorphism of TIMP-1. Patients with the T allele showed higher serum levels of TIMP-1 than patients without the T allele (*P *= 0.004). Multiple logistic regression analysis showed that the T allele was associated with higher mortality at 30 days (odds ratio = 2.08; 95% confidence interval = 1.06 to 4.09; *P *= 0.03). Survival analysis showed that patients with the T allele presented lower 30-day survival than patients without the T allele (χ^2 ^= 5.77; *P *= 0.016). We found an association between TIMP-1 levels and levels of MMP-9 (ρ = -0.19; *P *= 0.002), MMP-10 (ρ = 0.55; *P *<0.001), TNFα (ρ = 0.56; *P *<0.001), IL-10 (ρ = 0.48; *P *<0.001) and PAI-1 (ρ = 0.49; *P *<0.001).

**Conclusion:**

The novel findings of our study are that septic patients with the T allele in the 372 T/C genetic polymorphism of TIMP-1 showed higher serum TIMP-1 levels and lower survival rate. The determination of the 372 T/C genetic polymorphism of TIMP-1 thus has prognostic implications and could help in the selection of patients who may benefit from modulation of the MMP/TIMP balance.

## Introduction

Sepsis represents a systemic response of the immune system to infection that leads to high mortality and costs [1,2]. The pathophysiologic mechanisms of sepsis are not well known and a better understanding of these processes may allow unmasking of other mortality risk factors.

Matrix metalloproteinases (MMPs) are a family of zinc-containing endoproteinases implicated in the degradation and remodelling of the extracellular matrix. The regulation of MMP activity is carried out by tissue inhibitors of matrix metalloproteinases (TIMPs). MMPs have a role in normal physiological functions such as the menstrual cycle, morphogenesis, tissue remodelling and angiogenesis, and in diseases with abnormal extracellular matrix turnover, such as arthritis, tumour invasion, aneurysm formation and atherosclerosis [3]. The role of MMPs/TIMPs in sepsis remains unclear; however, the results of some studies indicate that MMPs facilitate the recruitment of leukocytes from the bloodstream to the site of infection for eradication of the pathogen by proteolysis of the basement membrane [4], and modulate inflammatory [4] and prothrombotic responses [5,6].

Higher circulating levels of TIMP-1 have been reported in nonsurviving septic patients than in surviving septic patients [7-9]. An association between some genetic polymorphisms of the X-linked TIMP-1 gene and the risk of developing certain diseases has been reported [10-23], the 372 T/C polymorphism being the variant most studied [10-16]. However, the relationship between genetic polymorphism of TIMP-1, circulating TIMP-1 levels and survival in patients with severe sepsis has not been examined.

The objective of this study was thus to determine whether there is an association between the 372 T/C genetic polymorphism of TIMP-1, serum levels of TIMP-1 and survival in patients with severe sepsis.

## Materials and methods

### Design and subjects

A multicentre, prospective, observational study was carried out in six Spanish ICUs. The study was approved by the Institutional Review Boards of the six hospitals: Hospital Universitario de Canarias (La Laguna, Santa Cruz de Tenerife, Spain), Hospital Universitario Nuestra Señora de Candelaria (Santa Cruz de Tenerife, Spain), Hospital Universitario Dr. Negrín (Las Palmas de Gran Canaria, Spain), Hospital Clínico Universitario de Valencia (Valencia, Spain), Hospital San Jorge (Huesca, Spain) and Hospital Insular (Las Palmas de Gran Canaria, Spain). Written informed consent was obtained from the patients or from the family members. All patients were Caucasian and suffered from severe sepsis. The diagnosis of severe sepsis was established according to the International Sepsis Definitions Conference [24].

Exclusion criteria were: age <18 years, pregnancy, lactation, HIV, white blood cell count <10^3^/mm^3^, solid or haematological tumours, or immunosuppressive, steroid or radiation therapy.

### Variables recorded

The following variables were recorded for each patient: sex, age, diabetes mellitus, site of infection, microorganism responsible, bloodstream infection, adequate empiric antimicrobial treatment, pressure of arterial oxygen/fraction of inspired oxygen, creatinine, bilirubin, leukocyte count, lactic acid, platelet count, International Normalised Ratio (INR), activated partial thromboplastin time (aPTT) and Acute Physiology and Chronic Health Evaluation (APACHE) II score [25]. Measurements of circulating levels of TIMP-1, MMP-9, MMP-10, TNFα, IL-10 and plasminogen activator inhibitor-1 (PAI-1), as well as the genetic determination of the TIMP-1 372 T/C polymorphism were carried out in peripheral blood. Survival at 30 days from ICU admission was the endpoint assessed.

### Determination of the 372 T/C genetic polymorphism of TIMP-1 (rs4898)

Genotyping was performed by PCR and restriction fragment length polymorphism analysis. DNA was prepared from 3 ml peripheral blood using proteinase K treatment, phenol-chloroform extraction and ethanol precipitation. About 100 ng DNA were used as the template in PCR using the primers 5'-GCACATCACTACCTGCAGTCT-3' and 5'-GAAACAAGCCCACGATTTAG-3', flanking the 372 T/C polymorphism (rs4898) of the TIMP-1 gene, and the temperature profile 94°C-55°C-72°C for 30 seconds each for 30 cycles. (C denotes a mutation introduced in the primer in order to generate a *Bss*SI restriction site (CACGAG) in the C allele, while the T allele remains uncut.) The amplified DNA was restricted with endonuclease *Bss*SI (New England Biolabs, Boston, MA, USA) for 2 hours at 37°C. The resulting DNA fragments were separated by gel electrophoresis in 2.5% agarose gel and visualised under ultraviolet light. In the absence of a *Bss*SI site, a fragment of 175 base pairs was detected (T allele), whereas fragments of 153 and 22 base pairs corresponded to the C allele. Genotyping was performed in a blinded manner.

### Determination of serum TIMP-1, MMP-9, MMP-10, TNFα, IL-10 and plasma PAI-1 levels

Blood samples were collected at ICU admission. Serum separator tubes were used to determine TIMP-1 concentration in serum; venous blood samples were taken and centrifuged within 30 minutes at 1,000×*g *for 15 minutes, and the serum was removed and frozen at -80°C until measurement.

TIMP-1, MMP-9 and MMP-10 assays were performed at the Atherosclerosis Research Laboratory of CIMA - University of Navarra (Pamplona, Spain). A specific ELISA (Quantikine^®^; R&D Systems, Abingdon, UK) was used in accordance with the manufacturer's instructions with a serum dilution of 1:100, 1:80 and 1:2, respectively. The interassay coefficient of variation was <8% (*n *= 20) and the detection limits for the assays were 0.15 ng/ml, 0.31 ng/ml and 78.1 pg/ml, respectively.

TNFα, IL-10 and PAI-1 assays were performed at the Laboratory Department of the Hospital Universitario de Canarias (La Laguna, Santa Cruz de Tenerife, Spain). TNFα and IL-10 determinations were performed by solid-phase chemiluminescent immunometric assays (Immulite^®^; Siemens Healthcare Diagnostics Products, Llanberis, UK). The interassay coefficients of variation were <6.5% (*n *= 20) and <9.9% (*n *= 40), respectively, and detection limits for the assays were 1.7 pg/ml and 1 pg/ml, respectively. PAI-1 antigen was assayed by specific ELISA (Imubind Plasma PAI-1 Elisa™; American Diagnostica, Inc., Stanford, CT, USA). This assay detects latent (inactive) and active forms of PAI-1 and PAI-1 complexes. The interassay coefficient of variation was <5% (*n *= 20) and the detection limit for the assay was 1 ng/ml.

### Statistical methods

This series of patients was a nonprobabilistic sample and the recruitment period was 18 months. This study included 192 patients from a previous publication [8]. Continuous variables are reported as medians and interquartile ranges. Categorical variables are reported as frequencies and percentages. Comparisons of continuous variables between groups were carried out using the Wilcoxon-Mann-Whitney test. Comparisons between groups on categorical variables were carried out with the chi-square test. The association between continuous variables was carried out using Spearman's rank correlation coefficient or Spearman's rho coefficient.

Thirty-day survival curves, using the T allele or non-T allele of the 372 T/C genetic polymorphism of TIMP-1, were represented using the Kaplan-Meier method and compared by log-rank test. Multivariate logistic regression analysis was applied to data from the whole sample and separately by sex in order to determine the independent contribution of the 372 T/C genetic polymorphism of TIMP-1, lactic acid levels, APACHE II score and sex to the prediction of mortality during the 30-day period. We analysed, both globally and separately by sex, the relationship between TIMP-1 levels and 30-day survival, controlling for lactic acid level and APACHE score.

Odds ratios (ORs) and 95% confidence intervals (CIs) were calculated as measures of the clinical impact of the predictor variables. Using linear regression modelling, we analysed the relationship between the 372 T/C genetic polymorphism of TIMP-1 and the infection site as independent variables and TIMP-1 levels as the dependent variable. *P *<0.05 was considered statistically significant. Statistical analyses were performed with SPSS 17.0 (SPSS Inc., Chicago, IL, USA) and NCSS 2000 (Jerry Hintze, Kaysville, UT, USA).

## Results

As shown in Table 1, a total of 275 patients with severe sepsis were included, 80 with genotype CC (or male hemizygous C), 55 with genotype CT and 140 with genotype TT (or male hemizygous T) of the 372 T/C genetic polymorphism of TIMP-1 (rs4898). The calculated frequencies for the C (0.393) and T (0.607) alleles in our sample were similar to those obtained in the Exome Sequencing Project cohort population (0.467 and 0.533, respectively) [26]. Since TIMP-1 is located on the × chromosome, men and women were considered separately to test for Hardy-Weinberg equilibrium among our genotypes. Using chi-square tests to compare expected and observed genotypes, we found no significant deviation from Hardy-Weinberg predictions. There were no significant differences between different genotypes in age, diabetes mellitus, site of infection, microorganism responsible, bloodstream infection, adequate empiric antimicrobial treatment, pressure of arterial oxygen/fraction of inspired oxygen, bilirubin, leukocyte count, INR, aPTT and APACHE II score. However, patients with the T allele showed higher serum creatinine and lactic acid levels, and lower platelet count and male sex. Besides, patients with the T allele of the 372 T/C genetic polymorphism of TIMP-1 showed higher serum levels of TIMP-1 (*P *= 0.004) and lower survival rate (*P *= 0.02) than patients without the T allele.

**Table 1:** Patient characteristics according to the 372 C/T genetic polymorphism of tissue inhibitor of matrix metalloproteinase-1

Characteristic	Without T allele (*n *= 80)	With T allele (*n *= 195)	*P *value
Gender (male)	70 (87.5)	111 (56.9)	<0.001
Age (years)	62 (53 to 72)	60 (48 to 69)	0.12
Diabetes mellitus	26 (32.5)	60 (30.8)	0.77
Myocardial infarction	7 (8.8)	11 (5.6)	0.42
Chronic obstructive pulmonary disease	12 (15.0)	26 (13.3)	0.70
Asthma	2 (2.5)	4 (2.1)	0.99
Site of infection			0.86
Respiratory	47 (58.8)	106 (54.4)	
Abdominal	23 (28.8)	57 (29.2)	
Neurological	1 (1.3)	4 (2.1)	
Urinary	2 (2.5)	12 (6.2)	
Skin	3 (3.8)	8 (4.1)	
Endocarditis	4 (5.0)	7 (3.6)	
Others	0 (0)	1 (0.5)	
Bloodstream infection	11 (13.8)	31 (15.9)	0.71
PaO_2_/FIO_2 _ratio	150 (103 to 248)	176 (107 to 262)	0.82
Creatinine (mg/dl)	1.00 (0.85 to 2.03)	1.40 (0.80 to 2.40)	0.02
Bilirubin (mg/dl)	0.80 (0.43 to 1.45)	0.90 (0.46 to 1.80)	0.64
Leukocytes (×10^3^/mm^3^)	14.6 (10.2 to 20.0)	15.0 (9.1 to 20.4)	0.99
Lactic acid (mmol/l)	1.80 (1.20 to 3.10)	2.30 (1.30 to 4.60)	0.02
Platelets (×10^3^/mm^3^)	227 (132 to 303)	165 (93 to 234)	0.007
INR	1.32 (1.06 to 1.55)	1.35 (1.14 to 1.58)	0.46
aPTT (seconds)	33 (29 to 44)	32 (28 to 41)	0.28
APACHE II score	19 (15 to 24)	20 (16 to 24)	0.41
TIMP-1 (ng/ml)	523 (355 to 707)	639 (444 to 880)	0.004
MMP-9 (ng/ml)	759 (389 to 1,224)	606 (275 to 1,022)	0.11
MMP-10 (pg/ml)	1706 (1,016 to 2,539)	1,795 (1,237 to 3,514)	0.12
TNFα (pg/ml)	27.8 (18.1 to 47.1)	31.8 (20.1 to 57.9)	0.37
IL-10 (pg/ml)	10.1 (5.6 to 55.4)	14.55 (6.0 to 60.5)	0.36
PAI-1 (ng/ml)	39.9 (25.2 to 63.4)	44.8 (24.6 to 71.5)	0.48
Survivors at 30 days	61 (76.3)	118 (60.5)	0.02

Data presented as number (percentage) or median (25th to 75th percentile). APACHE, Acute Physiology and Chronic Health Evaluation; aPTT, activated partial thromboplastin time; INR, International Normalised Ratio; MMP, matrix metalloproteinase; PaO_2_/FIO_2_, pressure of arterial oxygen/fraction inspired oxygen; PAI-1, plasminogen activator inhibitor-1; TIMP, tissue inhibitor of matrix metalloproteinase.

Thirty-day survival according to the different 372 T/C genetic polymorphism of TIMP-1 was analysed separately by sex. In men, we found higher survival in patients with the C allele than in those with the T allele (53/70 (75.7%) vs. 67/111 (60.4%); *P *= 0.04). In women, we did not find significant differences in survival between patients with C/C, C/T and T/T expression (8/10 (80%), 34/55 (61.8%) vs. 17/29 (58.6%); *P *= 0.47).

As shown in Table 2, we found higher TIMP-1 levels in nonsurvivors than in survivors globally; separately by sex and 372 T/C genetic polymorphism of TIMP-1, we found higher TIMP-1 levels in women and men with the T allele (Table 2).

**Table 2:** TIMP-1 levels in surviving and nonsurviving patients according to sex and 372 T/C genetic polymorphism

	Survival	Nonsurvival	*P *value
All patients	529 (355 to 706)	805 (510 to 1,027)	<0.001
Women			
C/C	436 (345 to 775)	662 (500 to 824)	0.43
C/T	583 (415 to 819)	862 (506 to 1,055)	0.03
T/T	465 (284 to 754)	742 (413 to 1,177)	0.046
Men			
C allele	500 (333 to 657)	696 (370 to 936)	0.11
T allele	557 (437 to 715)	814 (616 to 1,055)	<0.001

Data (ng/ml) presented as median (25th to 75th percentile). TIMP, tissue inhibitor of matrix metalloproteinase. **{AU Query: Confirm table caption, contents and footnote after editing to style}**

We found an association between TIMP-1 levels and MMP-9 (ρ = -0.19; *P *= 0.002), MMP-10 (ρ = 0.55; *P *<0.001), TNFα (ρ = 0.56; *P *<0.001), IL-10 (ρ = 0.48; *P *<0.001) and PAI-1 (ρ = 0.49; *P *<0.001) levels, INR (ρ = -0.42; *P *<0.001), aPTT (ρ = 0.29; *P *<0.001) and platelet count (ρ = -0.18; *P *= 0.002).

As shown in Table 3, multiple logistic regression analysis showed that the T allele of the 372 T/C genetic polymorphism of TIMP-1 was associated with higher mortality at 30 days (OR = 2.08; 95% CI = 1.06 to 4.09; *P *= 0.03). Separately by sex, we found a relationship between the T allele of the 372 T/C genetic polymorphism of TIMP-1 and survival in men, after controlling for lactic acid level and APACHE II score (OR = 1.44; 95% CI = 1.002 to 2.072; *P *= 0.049), but not in women (OR = 1.45; 95% CI = 0.57 to 3.64; *P *= 0.43).

**Table 3:** Multiple logistic regression analysis to predict 30-day mortality.

	Odds ratio	95% confidence interval	*P *value
First model including all patients			
Lactic acid	1.19	1.07 to 1.32	0.001
APACHE II	1.08	1.04 to 1.13	<0.001
Gender female	1.26	0.68 to 2.33	0.46
Presence of T allele^a^	2.08	1.06 to 4.09	0.03
Second model including only women			
Lactic acid	1.18	1.02 to 1.36	0.03
APACHE II	1.10	1.02 to 1.18	0.009
Presence of T allele^a^	1.45	0.57 to 3.64	0.43
Third model including only men			
Lactic acid	1.21	1.04 to 1.40	0.01
APACHE II	1.08	1.02 to 1.13	0.008
Presence of T allele^a^	1.44	1.002 to 2.072	0.049
Four model including all patients			
Lactic acid	1.13	1.02 to 1.27	0.03
APACHE II	1.08	1.03 to 1.13	<0.001
TIMP-1 levels	1.001	1.0002 to 1.002	0.008
Five model including only women			
Lactic acid	1.13	0.95 to 1.34	0.16
APACHE II	1.14	1.04 to 1.24	0.003
TIMP-1 levels	1.001	1.0002 to 1.003	0.048
Six model including only men			
Lactic acid	1.13	1.06 to 1.62	0.01
APACHE II	1.10	1.02 to 1.19	0.02
TIMP-1 levels	1.0001	0.999 to 1.001	0.48

APACHE, Acute Physiology and Chronic Health Evaluation; TIMP, tissue inhibitor of matrix metalloproteinase. ^a^T allele of the 372 T/C genetic polymorphism of TIMP-1.

As shown in Table 3, multiple logistic regression analysis showed that TIMP-1 levels were associated with higher mortality at 30 days after controlling for lactic acid level and APACHE II score (OR = 1.001; 95% CI = 1.0002 to 1.002; *P *= 0.008). Separately by sex, we found a relationship between TIMP-1 levels and survival in women, after controlling for lactic acid level and APACHE II score (OR = 1.001; 95% CI = 1.0002 to 1.003; *P *= 0.048), but not in men (OR = 1.0001; 95% CI = 0.999 to 1.001; *P *= 0.48).

Linear regression analysis showed that TIMP-1 levels were associated with the 372 T/C genetic polymorphism of TIMP-1 (regression coefficient = 114.4; 95% CI = 31.71 to 197.14; *P *= 0.007), but not with site of infection (regression coefficient = 105.7; 95% CI = -45.12 to 256.48; *P *= 0.17).

**Figure 1:**
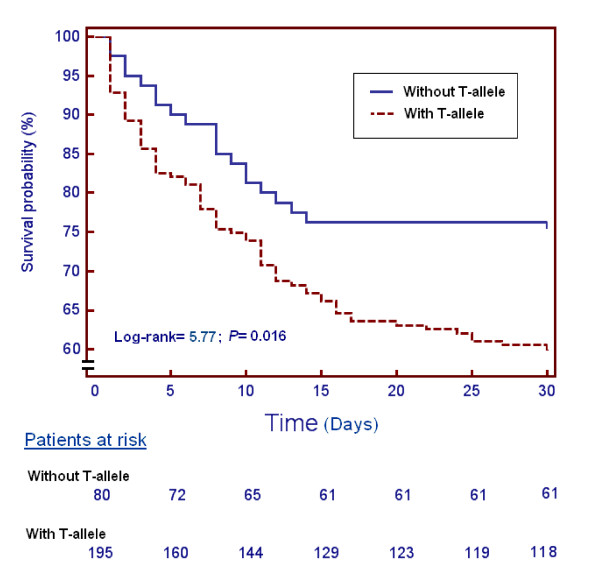
**Thirty-day survival according the T allele in the 372 C/T genetic polymorphism of TIMP-1**. Kaplan-Meier curves showing the cumulative proportion of survival patients at 30 days according to the presence or not of the T allele in the 372 C/T genetic polymorphism of tissue inhibitor of matrix metalloproteinase (TIMP)-1.

Survival analysis showed that patients with the T allele of the 372 T/C genetic polymorphism of TIMP-1 presented lower 30-day survival than those without the T allele (χ^2 ^= 5.77; *P *= 0.016) (Figure 1).

## Discussion

The novel findings of our study were that there is an association between the 372 T/C genetic polymorphism of TIMP-1 (rs4898), circulating levels of TIMP-1 and 30-day survival in patients with severe sepsis. Patients with the T allele in the 372 T/C genetic polymorphism of TIMP-1 showed higher serum TIMP-1 levels and lower survival rate.

Although the exact mechanism by which this synonymous SNP might affect function is unknown, the 372 T/C genetic polymorphism of TIMP-1 has been associated with increased risk of developing certain diseases [10-16]. However, an association between the 372 T/C genetic polymorphism of TIMP-1 and survival in patients with severe sepsis has not been previously reported. The results of the different studies are controversial; some found that the C allele was associated with higher risk of certain diseases [10-13] while others showed that the T allele was associated with higher risk [14-16]. In the present study, septic patients with the T allele had a lower rate of survival at 30 days.

A previous study showed that humans carrying the T allele in the 372 T/C genetic polymorphism of TIMP-1 had increased susceptibility to Crohn's disease and presented lower levels of TIMP-1 in surgically resected macroscopically inflamed tissue [15]. In our study, however, we found that septic patients with this T allele had a lower survival rate and showed higher serum levels of TIMP-1. This is consistent with reports of higher circulating levels of TIMP-1 in nonsurviving septic patients than in surviving septic patients [7-9].

There is a relationship between coagulation and inflammation response in sepsis [27-29] and it is possible that MMPs/TIMPs play a role in that response. In our series, septic patients carrying the T allele showed significantly higher serum TIMP-1 levels and lower survival rate. The reason for this is not clear; TIMP-1 forms noncovalent bimolecular complexes with the active and latent forms of MMP-9, and reduces MMP-9 activity [3,4]. Some studies have reported that MMP-9 inhibits platelet aggregation [30,31], that there is a positive correlation between TIMP-1 and PAI-1 [32,33] and that there is a positive correlation between TIMP-1 and plasma homocysteine [32]. We found, for the first time in septic patients, a positive association between TIMP-1 levels and PAI-1 levels and aPTT, and a negative association between TIMP-1 levels and the INR and platelet count - these data suggest that these patients had a higher anti-fibrinolytic state and activation of coagulation. We found that septic patients carrying the T allele in the 372 T/C genetic polymorphism of TIMP-1 had higher TIMP-1 levels than those without it. We therefore believe that septic patients carrying the T allele in the 372 T/C genetic polymorphism of TIMP-1 could have a prothrombotic/antifibrinolytic profile, responsible for capillary thrombosis, multiple organ dysfunction and, finally, early death.

The 372 T/C genetic polymorphism of TIMP-1 is located on the × chromosome, which means that men have the C or T allele, and women have C/C, C/T or T/T. This aspect is important since there are differences in the findings according to patient gender. On the one hand, globally, we found that patients without the T allele showed better 30-day survival than those with the T allele. Separately by sex, men without the T allele had better 30-day survival than those with the T allele but no differences in survival were found between women patients with C/C, C/T and T/T alleles. On the other hand, globally, we found that nonsurvivors showed higher TIMP-1 levels than survivors. Stratified by sex, nonsurviving women with C/T and T/T and nonsurviving men with the T allele had higher TIMP-1 levels than survivors; however, we did not find differences in TIMP-1 levels between survivors and nonsurvivors in women with C/C or men with the C allele. Taken together, our results indicate an association between the T allele and TIMP-1 levels, and that higher TIPM-1 levels increased early mortality.

A noteworthy finding of the present study was that TIMP-1 levels in both surviving and nonsurviving patients were lower than those described in other series [7,9]; however, the levels were significantly higher in nonsurvivors than in survivors in all studies.

From a therapeutic perspective, modulators of MMP/TIMP activity have been used successfully in septic animal models, reducing TIMP-1 and improving the prognosis [34,35]. Determination of the 372 T/C genetic polymorphism of TIMP-1 could thus help in the selection of patients who may benefit from modulation of MMP/TIMP activity.

The present study has certain limitations. First, the sample size was relatively small; nevertheless, our nonprobabilistic sample was large enough to be able to show an association between the 372 T/C genetic polymorphism of TIMP-1 and 30-day survival. Second, it could be interesting to determine other SNPs of TIMP-1; however, we tested rs4898 because this SNP has previously been associated with other diseases [10-16]. rs4898 is a synonymous coding SNP, but it is in strong linkage disequilibrium with other TIMP-1 polymorphisms, as documented in the HapMap database for European Community populations [26]. The rs4898 SNP can thus be used as a tag SNP for the region of interest. It is possible that this rs4898 SNP is linked to other SNPs associated with the same effect, as was previously proposed in a study by Skarmoutsou and colleagues to explain the association between this polymorphism and systemic sclerosis [16]. Third, we did not determine the rs4898 SNP in healthy control subjects; however, the objective of our study was not to determine the association between the occurrence of sepsis and the polymorphism, but rather the association between sepsis survival and the polymorphism.

## Conclusion

The novel findings of our study are that septic patients with the T allele in the 372 T/C genetic polymorphism of TIMP-1 showed higher serum TIMP-1 levels and lower survival rate. Determination of the 372 T/C genetic polymorphism of TIMP-1 thus has prognostic implications and could help in the selection of patients who may benefit from modulation of the MMP/TIMP balance.

## Key messages

• Septic patients with T allele in the 372 T/C genetic polymorphism of TIMP-1 showed higher serum TIMP-1 levels and lower survival rate

• The determination of the 372 T/C genetic polymorphism of TIMP-1 has prognostic implications and could help in the selection of patients who may benefit from modulation of the MMP/TIMP balance.

## Abbreviations

APACHE: Acute Physiology and Chronic Health Evaluation; aPTT: activated partial thromboplastin time; INR: International Normalised Ratio; CI: confidence interval; ELISA: enzyme-linked immunosorbent assay; MMP: matrix metalloproteinase; OR: odds ratio; PAI-1: plasminogen activator inhibitor-1; PCR: polymerase chain reaction; SNP: single nucleotide polymorphism; TIMP: tissue inhibitor of matrix metalloproteinase; TNF: tumour necrosis factor.

## Competing interests

The authors declare that they have no competing interests.

## Authors' contributions

LLo conceived, designed and coordinated the study, participated in acquisition of data, and drafted the manuscript. MM, JS-V, JB, LLa and CD participated in acquisition of data. AJ interpreted the data. FP and ES carried out the molecular genetic studies. JMB-L, JAP, JO and JAR carried out the immunoassays. All authors read and approved the final manuscript.
